# Complications, Adverse Drug Events, High Costs, and Disparities in Multisystem Inflammatory Syndrome in Children vs COVID-19

**DOI:** 10.1001/jamanetworkopen.2022.44975

**Published:** 2023-01-05

**Authors:** William Encinosa, Kyung Moon, Jessica Figueroa, Youssef Elias

**Affiliations:** 1 Agency for Healthcare Research and Quality, Rockville, Maryland; 2 McCourt School of Public Health, Georgetown University, Washington, DC; 3 Division of Blood Diseases and Resources, National Heart, Lung, and Blood Institute, National Institutes of Health, Bethesda, Maryland; 4 Now with Bacteriology and Mycology Branch, Division of Microbiology and Infectious Diseases, National Institute of Allergy and Infectious Diseases, National Institutes of Health, Bethesda, Maryland; 5 Johns Hopkins Carey Business School, Baltimore, Maryland; 6 Department of Vascular and Interventional Radiology, Carle Foundation Hospital, Carle Illinois College of Medicine, Urbana

## Abstract

**Importance:**

Multisystem inflammatory syndrome in children (MIS-C) causes severe inflammation of multiple organ systems after SARS-CoV-2 infection. During the pandemic, surveillance reporting of MIS-C was voluntary, with likely underreporting. For a rare syndrome like MIS-C, numerous data are needed to explore the disease in greater detail.

**Objective:**

To use large all-payer billing data and the new *International Statistical Classification of Diseases and Related Health Problems, Tenth Revision, Clinical Modification* (*ICD-10-CM*) code for MIS-C to compare outcomes across MIS-C and COVID-19 over all 4057 hospitals in 31 states.

**Design, Setting, and Participants:**

A retrospective cross-sectional study of all COVID-19 and MIS-C hospitalizations in individuals younger than 21 years from 31 states was conducted, using Agency for Healthcare Research and Quality 2021 Healthcare Cost and Utilization Project data. Analyses were conducted from February 1 to October 20, 2022.

**Main Outcomes and Measures:**

Fifty complications, adverse medication events, costs, and the Social Vulnerability Index.

**Results:**

There were 4107 individuals with MIS-C (median age, 9 [IQR, 5-13] years; 2443 [59.5%] male; 1384 [38.1%] White) and 23 686 individuals with COVID-19 without MIS-C (median age, 15 [IQR, 5-18] years; 12 878 [54.4%] female; 4605 [44.1%] White), with 1.48 (95% CI, 1.35-1.62) MIS-C hospitalizations per 100 000 children per month, ranging from 0.97 hospitalizations per 100 children for White and 1.99 hospitalizations per 100 children for Black children. Outcomes worsened as the number of organ system dysfunctions increased from 2 to 8 organs. Deaths associated with MIS-C increased from less than 1% to 5.8% (95% CI, 3.3%-8.4%) and from less than 1% to 17.2% (95% CI, 11.7%-22.7%) for COVID-19 (*P* = .001). Adverse medication events associated with MIS-C increased from 4.9% (95% CI, 3.8%-6.0%) to 17.8% (95% CI, 13.7%-22.0%) and from 1.2% (95% CI, 1.0%-1.3%) to 13.4% (95% CI, 8.4%-18.3%) for COVID-19. The median length of stay for MIS-C increased from 4 (IQR, 2-5) to 8 (IQR, 5-12) days and from 3 (IQR, 2-5) to 16 (IQR, 7-23) days for COVID-19. Median costs for MIS-C increased from $16 225 (IQR, $9244-$26 822) to $53 359 (IQR, $35 920-$86 882) and from $6474 (IQR, $3741-$12 103) to $98 643 (IQR, $30 675-$204 956) for COVID-19. The percentage of MIS-C cases that were in Black children doubled from 16.2% to 31.7% (*P* = .001) as organ dysfunction increased, remaining unchanged with COVID-19. Hospital stays for MIS-C increased by 1 day (*P* = .01) for Black patients compared with White patients, with Black patients moving from the bottom to top quartile of socioeconomic vulnerability, with no disparity with COVID-19.

**Conclusions and Relevance:**

In this cross-sectional study, MIS-C was more common and severe than previously reported, with more racial disparities in outcomes than were seen in patients with COVID-19. The findings of this study suggest that relying on mean outcomes for MIS-C from past studies can be misleading, since outcomes and disparities varied widely with the number of multiorgan dysfunctions.

## Key Points

**Question:**

Did outcomes vary by the number of organ systems affected in multisystem inflammatory syndrome in children (MIS-C) in 2021?

**Findings:**

In this cross-sectional study of 4107 MIS-C hospitalizations, as the number of organ systems affected increased from 2 to 6 or more, mortality increased from 1% to 6%, length of stay doubled from 4 to 8 days, adverse medication events increased from 5% to 18%, and the percentage of patients with MIS-C who were Black doubled from 16% to 32%. All of these increases were statistically significant.

**Meaning:**

The findings of this study suggest that future efforts should focus on how to prevent MIS-C from progressing to multiple organ system dysfunction.

## Teaser

This cross-sectional study compares the incidence and factors associated with outcomes of multisystem inflammatory syndrome in children vs COVID-19.

## Introduction

After several waves of COVID-19, attention has now turned to understanding post–COVID-19 condition, which has been termed long COVID. For children, the most serious post-COVID complication has been multisystem inflammatory syndrome in children (MIS-C), a relatively novel hyperinflammatory syndrome that customarily arises approximately 1 month after a SARS-CoV-2 infection, sometimes resulting in cardiac complications in previously healthy children.^1,2,3,4,5,6,7,8,9,10,11,12,13,14,15^ Since mid-May 2020, the US Centers for Disease Control and Prevention (CDC) has been tracking case reports of MIS-C.

However, since the CDC surveillance of MIS-C relies on hospitals voluntarily reporting to state health departments, we do not yet have a complete picture of MIS-C across the full spectrum of US hospitals. Most CDC studies on MIS-C complications have been limited to at most 66 hospitals across 31 states, with studies on incidence having up to 1700 MIS-C cases.^15,16,17,18^ For a rare syndrome such as MIS-C, this small set of data poses a challenge to exploring the disease in greater detail. However, a new *International Statistical Classification of Diseases, 10th Revision, Clinical Modification* (*ICD-10-CM*) diagnosis code for MIS-C was established in 2021 for reimbursement purposes. In this study, we used this new coding to investigate MIS-C across 4057 hospitals in 31 states (77% of the US population). With these novel and more complete data that include 4107 MIS-C cases, we are able for what is, to our knowledge, the first time to provide a more complete picture of MIS-C across the US. Since a feature of MIS-C is multiorgan dysfunction, our first goal was to examine how death, adverse medication events (AMEs), length of stay, and costs vary in both MIS-C and COVID-19 by the number of organ systems affected. The second goal was to identify racial and ethnic disparities in these outcomes for MIS-C vs COVID-19. The third goal was to investigate how these racial and ethnic disparities varied across the CDC Social Vulnerability Index (SVI) for MIS-C and COVID-19 to better understand the socioeconomic factors associated with the racial and ethnic disparities in MIS-C.

## Methods

### MIS-C and COVID-19 Data

We used the Agency for Healthcare Research and Quality (AHRQ) Healthcare Cost and Utilization Project (HCUP) 2021 quarterly inpatient data for 31 states reporting data as of October 20, 2022. Analyses were conducted from February 1 to October 20, 2022. Details of the 31 state data files are found in eAppendix 1 in Supplement 1. These data report all patient discharges from the 4057 community hospitals in these 31 states, which represent 77% of the US population.^18,19^ We subset to age younger than 21 years and created 2 subsamples: MIS-C discharges and COVID-19 discharges without MIS-C. We used the CDC *ICD-10-CM* coding instructions to identify patients with MIS-C (code M35.81) either as currently having COVID-19 (code U07.1) or COVID-19 sequelae (code U07.1 with code B94.8) or having a history of COVID-19 (code Z86.16) or known or suspected exposure to COVID-19 (code Z20.822).^20^ We compared the following clinical and usage outcomes across MIS-C and COVID-19. This study was approved by the institutional review board of the AHRQ and followed the Strengthening the Reporting of Observational Studies in Epidemiology (STROBE) reporting guideline. The need for patient informed consent was waived owing to the use of deidentified patient data according to the Department of Health and Human Services (45 CFR §46).

### Clinical Outcomes

We examined 2 clinical outcomes: inpatient death and AMEs during treatment, particularly with glucocorticoids and immunoglobulin (the 2 main treatments for MIS-C), coded by the hospitals with *ICD-10-CM* AME codes. Adverse medication events for glucocorticoids (code T38.0X5) involve hyperglycemia, myopathy, and changes in mood. Adverse medication events for immunoglobulin (code T50.Z15) include headache, nausea, and vomiting. Other AMEs examined were for antibiotics, diuretics, antihypertensives, and anticoagulants (eTable 1 in Supplement 1 provides the codes).

### Usage Outcomes

We examined 2 usage outcomes: length of stay and costs. The hospital costs were computed by applying the 2021 hospital-level Centers for Medicaid & Medicare Healthcare Cost Report Information System cost-to-charge ratios to charges in HCUP. Costs were then adjusted with the Centers for Medicaid & Medicare Wage Index. Costs represent the expenses incurred in the production of hospital services, such as wages, supplies, and utility, but exclude physician fees. For the disparities analysis, we also created 2 binary variables: 10 or more days in the hospital and costs greater than $44 000 (top 25th percentiles).

### Number of Organ Systems With Complications

Since MIS-C and COVID-19 can have a wide range of severity, we further compared clinical and usage outcomes by stratifying both the MIS-C and COVID-19 subsamples by the number of organ systems affected by complications. Following the Feldstein et al^10^ description of MIS-C complications, we examined more than 50 complications comprising 8 different organ dysfunction categories related to MIS-C hospitalizations (*ICD-10-CM* codes can be found in eTable 1 in Supplement 1): (1) cardiac, (2) respiratory, (3) neurological, (4) hematologic (5) gastrointestinal, (6) musculoskeletal, (7) kidney, and (8) mucocutaneous complications. We stratified MIS-C and COVID-19 into 5 categories: 0 to 2 systems, 3 systems, 4 systems, 5 systems, and 6 to 8 systems affected by complications.

### Statistical Analysis

The first goal was to examine how the clinical and usage outcomes vary in both MIS-C and COVID-19 by the number of organ systems affected. All 2-sided *P* values for differences between MIS-C and COVID-19 were generated from linear regression analyses or quantile regressions for testing medians of the usage variables. A 2-sided *P* ≤ .05 was considered statistically significant.

The second goal was to identify racial and ethnic disparities in complications, clinical outcomes, and usage outcomes for both MIS-C vs COVID-19. For binary variables this was done by fitting a Poisson regression with robust variance estimates to generate risk ratios.

The third goal was to examine how racial and ethnic disparities varied across quartiles of the SVI for MIS-C and COVID-19 in hospitalization incidence rates and length of stay. The SVI of the patient’s county is from the CDC and represents 15 social factors, including high poverty, low percentage of vehicle access, or crowded households, which affect that community’s ability to prevent human distress during the pandemic.^21^ Quantile regressions were used to analyze disparities in median length of stay across the SVI.

All regressions were conducted using Stata/MP, version 17 (StataCorp LLC), and, following Feldstein et al,^13^ covariates were age (5 bins), sex, race and ethnicity, and number of chronic conditions. Children were identified as Asian or Pacific Islander, Black, Hispanic, non-Hispanic White, or other (including Native American or other as reported by the Healthcare Cost and Utilization Project). Race and ethnicity data collection was a requirement of the funding body. Chronic conditions were coded using the AHRQ Elixhauser comorbidity software.^22^ Race and ethnicity was identified by hospitals and was missing for 8.5% of the sample, with most missing data occurring in Georgia. No missing race and ethnicity imputations were made; however, all regression analyses controlled for missing race and ethnicity. Race and ethnicity proportions are given for the subsample with nonmissing race.

## Results

### Demographic Characteristics of Individuals Hospitalized With MIS-C

As noted in Table 1, for the 31 states in our 2021 sample comprising 77% of the US population, we observed 4107 billed MIS-C hospitalizations (median age, 9 [IQR, 5-13] years; 2443 [59.5%] male; 1664 [40.5%] female; 881 [24.3%] non-Hispanic Black [hereafter, Black]). Patients with COVID-19 without MIS-C included 23 686 individuals (median age, 15 [IQR, 5-18] years; 12 878 [54.4%] female; 10 808 (45.6%) male; 4472 [20.5%] Black). The share of hospitalized children with MIS-C who were Black was higher than in those with COVID-19 (MIS-C: 24%; 95% CI, 23%-26% vs COVID-19: 21%; 95% CI, 20%-21%; *P* = .001). In contrast, the share of hospitalizations for children who were Hispanic did not differ significantly between MIS-C and COVID-19. Hospitalizations for MIS-C occurred in more male and younger individuals, in higher income areas, and with fewer chronic conditions than in children with COVID-19. Medicaid paid for 51% of the hospitalizations for MIS-C and 59% of those for COVID-19 (*P* = .001).

**Table 1.** Descriptive Statistics of 2021 MIS-C and Pediatric COVID-19 Hospitalizations in 31 States^a^

Characteristic	No. (%)		*P* value^b^
	MIS-C	COVID-19 without MIS-C	
No.	4107	23 686	<.001
No. per 100 000 children per month in Q1, mean (95% CI)	1.48 (1.35-1.62)	6.04 (5.45-6.64)	<.001
No. of hospitals	362	1921	<.001
Teaching hospital	4022 (97.9)	20 663 (87.2)	<.001
Transferred in	1108 (27.0)	4267 (18.0)	<.001
Currently has COVID-19	1245 (30.3)	23 686 (1.00)	<.001
Sex			
Male	2443 (59.5)	10 808 (45.6)	<.001
Female	1664 (40.5)	12 878 (54.4)	
Age, median (IQR)	9 (5-13)	15 (5-18)	<.001
No. of chronic conditions, mean (95% CI)	0.24 (22.5-25.6)	0.46 (45.6-47.4)	<.001
Social vulnerability index (range, 0-1), mean (95% CI)	0.55 (53.7-55.3)	0.54 (53.8-54.5)	.14
Low income	1274 (31.0)	8404 (35.5)	<.001
Length of stay, median (IQR), d	5 (3-7)	2 (2-5)	<.001
Inpatient costs, median (IQR), $	25 644 (14 353-44 229)	7517 (4114-15 328)	<.001
Medicaid	2065 (50.3)	13 830 (58.3)	<.001
Race and ethnicity^c^			
Asian or Pacific Islander, non-Hispanic	128 (3.5)	602 (2.8)	.03
Black, non-Hispanic	881 (24.3)	4472 (20.5)	<.001
Hispanic	988 (27.2)	5815 (26.7)	.48
White, non-Hispanic	1384 (38.1)	9605 (44.1)	<.001

Abbreviations: MIS-C, multisystem inflammatory syndrome in children; Q1, first quarter.

^a^ All MIS-C and COVID-19 discharges in individuals younger than 21 years over all 4057 hospitals in 31 states.

^b^ *P* value: COVID-19 compared with MIS-C.

^c^ Race percentages calculated on 25 431 children with nonmissing race. Race and ethnicity data missing for 8.5% of the sample.

### Incidence of MIS-C Hospitalizations

Restricting our sample to the 2715 children with MIS-C in quarter 1 (Q1), the MIS-C hospitalization rate was 1.48 (95% CI, 1.35-1.62) per 100 000 children per month. The overall incidence varied 2-fold by race: 0.97 for non-Hispanic White (hereafter, White) children and 1.99 for Black children (*P* = .01). This is a larger disparity than found with COVID-19, where the incidence varied from 4.4 for White children to 6.6 for Black children (*P* = .01). These incidence rates varied even further across areas with increased social vulnerability, as measured with the CDC SVI. In eFigure 1 in Supplement 1, moving from the lowest to highest quartile of the SVI, the COVID-19 incidence for Hispanic children increased from 5.5 to 9.8 (*P* = .002), from 5.6 to 7.9 for Black children (*P* = .01), and from 3.9 to 6.4 for White children (*P* = .001). For MIS-C, only the incidence in Hispanic children varied significantly with the SVI, from 0.9 to 1.8 (*P* = .001).

### Hospital Use

While we examined all 4057 community hospitals in the 31 states, only 1921 of these hospitals diagnosed COVID-19 in pediatric patients. A total of 362 of the hospitals diagnosed MIS-C, with 97.9% of these being teaching hospitals, compared with 87.2% for patients with COVID-19. A total of 27.0% of the children with MIS-C were transferred from other hospitals, compared with 18.0% of those with COVID-19. The median length of stay for MIS-C was 5 (IQR, 3-7) days compared with 2 (IQR, 2-5) days for COVID-19 (*P* < .001). Median hospital costs for MIS-C were $25 644 (IQR, $14 353-$44 229) per visit, compared with $7517 (IQR, $4114-$15 328) for COVID-19 (*P* < .001). In Q1 of 2021, we estimated the total hospital costs of MIS-C to be $97 million for these 31 states, as opposed to $194 million for COVID-19.

### Clinical Outcomes

As noted in Table 2, the mean inpatient death rate did not differ significantly for MIS-C (8 deaths per 1000 hospitalizations) and COVID-19 (9 deaths per 1000 hospitalizations). Patients with MIS-C had significantly more AMEs, 9.8%, compared with 2.1% for COVID-19 (*P* < .001). Most of these adverse events were due to glucocorticoids, with 7.2% of patients with MIS-C developing adverse glucocorticoid events, as opposed to 1.3% for patients with COVID-19. The other major AMEs occurred with immunoglobulin: 1.5% in patients with MIS-C patients and none in patients with COVID-19. Another 1.5% of patients with MIS-C had other AMEs (eg, antibiotics, anticoagulants) compared with 0.8% of patients with COVID-19.

**Table 2.** MIS-C and COVID-19 Complication Rates^a^

Outcomes	No. (%)		*P* value^b^
	MIS-C	Pediatric COVID-19 without MIS-C	
No. of organs affected (95% CI)	3.1 (3.0-3.2)	1.5 (1.4-1.5)	<.001
Inpatient death	34 (0.8)	215 (0.9)	.62
Organ system complications^c^			
Cardiovascular			
Any	1672 (40.7)	1064 (4.5)	<.001
Coronary artery aneurysm	226 (5.5)	24 (0.1)	<.001
Myocarditis	486 (11.8)	78 (0.3)	<.001
Pericarditis/perieffusion	367 (8.9)	135 (0.6)	<.001
Shock	821 (20.0)	379 (1.6)	<.001
Respiratory	1222 (29.8)	5995 (25.3)	<.001
Neurological	195 (4.7)	1284 (5.4)	.07
Hematologic	1908 (46.5)	2703 (11.4)	<.001
Kidney failure	934 (22.7)	1350 (5.7)	<.001
Gastrointestinal	1813 (44.2)	5165 (21.8)	<.001
Musculoskeletal	137 (3.3)	320 (1.4)	<.001
Mucocutaneous	1135 (27.6)	355 (1.5)	<.001
Adverse medication events^c^			
Any	403 (9.8)	498 (2.1)	<.001
Glucocorticoids	296 (7.2)	308 (1.3)	<.001
Immunoglobulin	62 (1.5)	0	<.001
Other (antibiotics, diuretics, antihypertensives)	62 (1.5)	190 (0.8)	<.001
Observations	4107	23 686	NA

Abbreviations: MIS-C, multisystem inflammatory syndrome in children; NA, not applicable.

^a^ All MIS-C and COVID-19 discharges in individuals younger than 21 years over 4057 hospitals in 31 states.

^b^ *P* value: COVID-19 compared with MIS-C.

^c^ Complications and adverse events are defined in eAppendix 2 in Supplement 1.

### Outcome Variation by the Number of Organ Systems With Complications

As noted in Table 2, patients with MIS-C had more organ systems affected, 3.1 (95% CI, 3.0-3.2) vs 1.5 (95% CI, 1.4-1.5) for COVID-19 (*P* < .001). Individually, MIS-C was more severe than COVID-19 for 7 of the 8 complications (all but neurological, where there was no significant difference). The most common complications for MIS-C were hematologic, as opposed to respiratory complications for COVID-19.

In Table 3, we see that 8% of patients with MIS-C had 6 or more of the 8 organ systems in Table 2 affected by complications, compared with 1% of patients with COVID-19. Outcomes worsened as the number of organ system dysfunctions increased from 0 to 2 to 6 to 8 organs. Inpatient death for MIS-C increased from less than 1% to 5.8% (95% CI, 3.3%-8.4%), and from less than 1% to 17.2% (95% CI, 11.7%-22.7%) for COVID-19. Adverse medication events for MIS-C increased from 4.9% (95% CI, 3.8%-6.0%) to 17.8% (95% CI, 13.7%-22.0%) and from 1.2% (95% CI, 1.0%-1.3%) to 13.4% (95% CI, 8.4%-18.3%) for COVID-19. Median length of stay doubled from 4 (IQR, 2-5) to 8 (IQR, 5-12) days for MIS-C and tripled from 3 (IQR, 2-5) to 16 (IQR, 7-23) days for COVID-19. Median costs increased from $16 225 (IQR, $9244-$26 822) to $53 359 (IQR, $35 920-$86 882) for MIS-C and from $6474 (IQR, $3741-$12 103) to $98 643 (IQR, $30 675-$204 956) for COVID-19.

**Table 3.** Distribution of Outcomes by Number of Organ System Dysfunctions^a^

Outcome	No. of organ systems affected^b^				
	0-2	3	4	5	6-8
**Patients, by disease**					
MIS-C, No. (%)	1515 (37)	913 (22)	852 (21)	502 (12)	325 (8)
COVID-19, No. (%)	19 149 (81)	2616 (11)	1246 (5)	488 (2)	187 (1)
**Inpatient death, No. (%)**					
MIS-C	<11^c^	<11^c^	<11^c^	<11^c^	19 (5.8)
COVID-19	19 (0.1)	45 (1.7)	62 (5.0)	57 (11.7)	32 (17.2)
*P* value^d^	.69	.002	.001	.001	.001
**Inpatient days, median (IQR)**					
MIS-C	4 (2-5)	5 (3-7)	6 (4-8)	7 (5-10)	8 (5-12)
COVID-19	3 (2-5)	4 (2-8)	6 (3-13)	9 (4-19)	15.5 (7-23)
*P* value^d^	.001	.001	1.0	.002	.001
**Inpatient costs, median (IQR), $**					
MIS-C	16 225 (9244-26 822)	25 043 (15 008-39 465)	33 152 (21 063-50 673)	40 170 (25 627-66 565)	53 359 (35 920-86 882)
COVID-19	6474 (3741-12 103)	13 433 (7006-29 221)	21 933 (9485-60 697)	39 065 (16 353-106 307)	98 643 (30 675-204 956)
*P* value^d^	.001	.001	.001	.79	.001
**Adverse medication events, No. (%)**					
MIS-C	74 (4.9)	87 (9.5)	107 (12.6)	77 (15.3)	58 (17.8)
COVID-19	230 (1.2)	123 (4.7)	81 (6.5)	36 (7.4)	25 (13.4)
*P* value^d^	.001	.001	.001	.001	.19
**Black, non-Hispanic, No. (%)^e^**					
MIS-C	246 (16.2)	195 (21.3)	181 (21.2)	156 (31.1)	103 (31.7)
COVID-19	3540 (18.5)	561 (21.4)	238 (19.1)	92 (18.9)	41 (21.9)
*P* value^d^	.03	.96	.23	.001	.02

Abbreviation: MIS-C, multisystem inflammatory syndrome in children.

^a^ All MIS-C and COVID-19 discharges in individuals younger than 21 years over 4057 hospitals in 31 states.

^b^ Organ system complications are the 8 complication groups listed in Table 2; defined in eAppendix 2 in Supplement 1.

^c^ Masked due to cell size less than 11.

^d^ *P* value: COVID-19 compared with MIS-C.

^e^ Race calculated on 25 431 children with nonmissing race and ethnicity. Race and ethnicity data missing for 8.5% of the sample.

### Racial Disparities in Outcomes

As reported in Table 3, the percentage of MIS-C cases that were in Black children doubled from 16.2% to 31.7% (*P* = .001) as the number of organs affected increased from 0 to 2 to 6 to 8. In contrast, the percentage of COVID-19 cases in Black children had a nonsignificant change over the number of organs (from 18.5% to 21.9%; *P* = .23).

**Figure 1.**
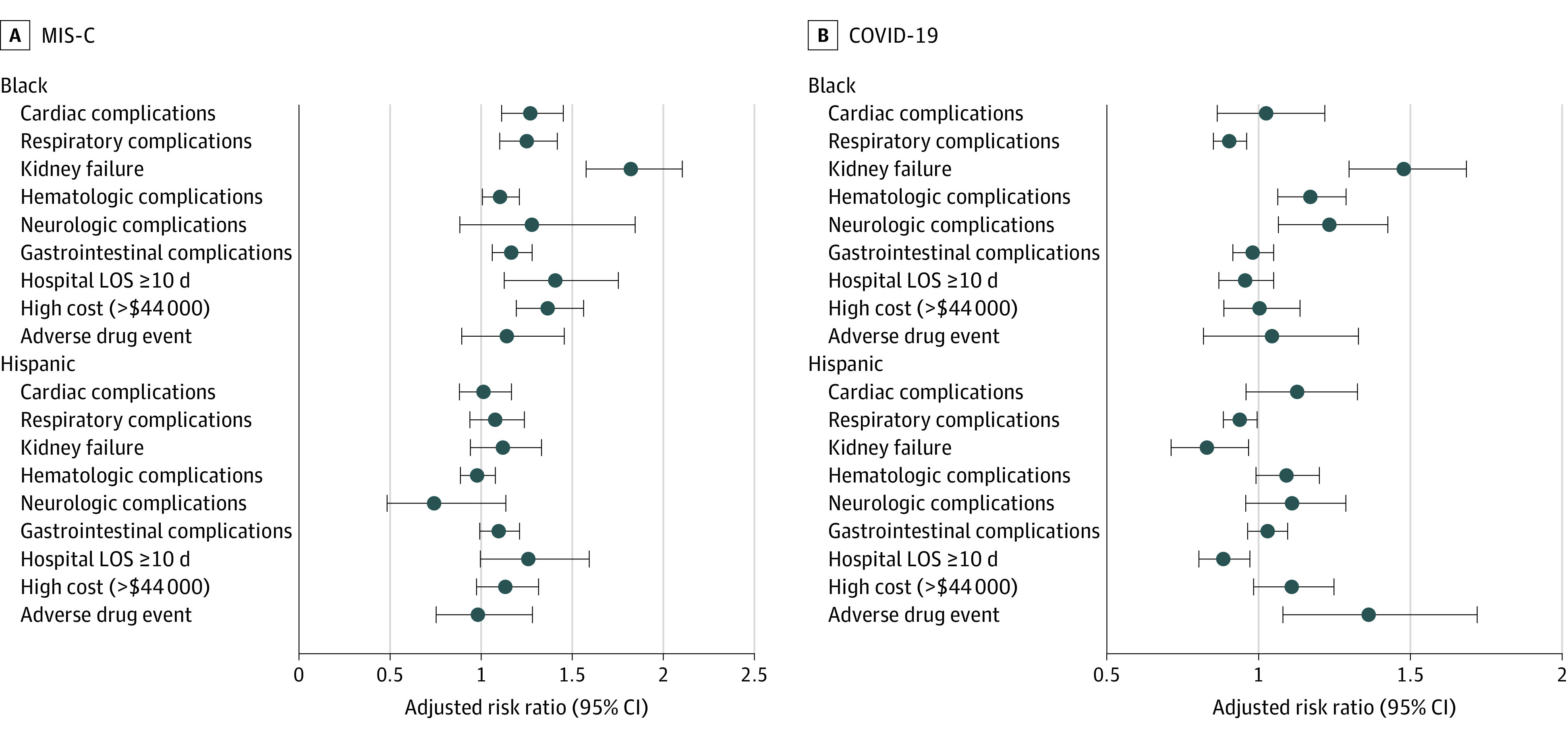
Racial and Ethnic Disparities in Risk-Adjusted Outcomes Disparities in outcomes in patients with multisystem inflammatory syndrome in children (MIS-C) (A) vs COVID-19 (B). Whiskers represent the 95% CI. LOS indicates length of stay.

Figure 1 depicts racial and ethnic disparities examined in risk-adjusted outcomes. A common pattern for both Black and Hispanic children was that they had lower relative risks of respiratory complications than White children with COVID-19 but greater relative risks of such with MIS-C. With MIS-C, Black children had higher relative risks than White children for 7 outcomes compared with only 3 in those with COVID-19. For MIS-C, the COVID-19 disparity in neurological complications for Black children disappeared, replaced by disparities in cardiac, respiratory, and gastrointestinal complications, as well as higher relative risks of staying 10 days or more in the hospital and incurring excessive costs (>$44 000) compared with White children, along with continuing disparities in hematologic and kidney complications. There were no disparities in death.

With COVID-19, Hispanic children had higher relative risks of an AME compared with White children. This risk, as well as their lower risk of having high costs, long stays, and kidney complications with COVID-19, disappeared (or reversed) in Hispanic children with MIS-C. Overall, Hispanic children had no significant disparities in outcomes with MIS-C except for higher risks of a long stay.

### Socioeconomic Variation in Racial and Ethnic Disparities

**Figure 2.**
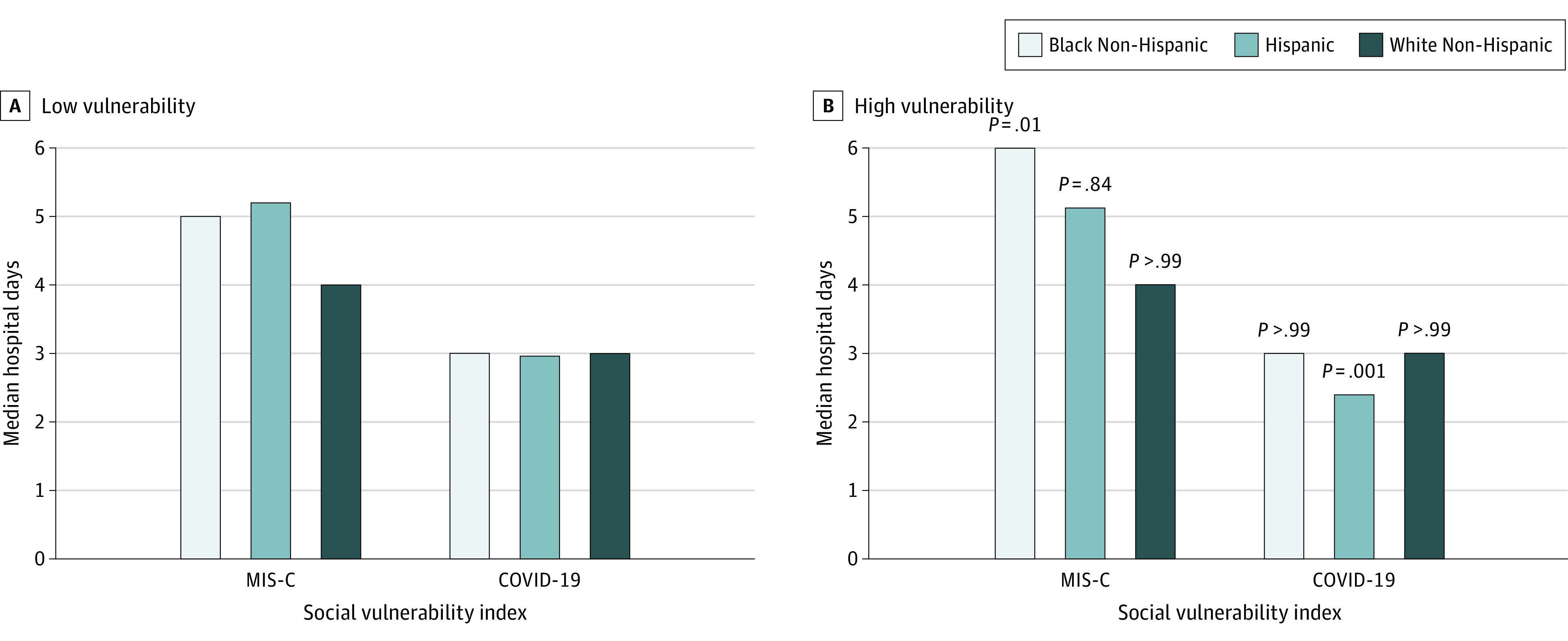
Socioeconomic and Racial and Ethnic Disparities in Risk-Adjusted Median Hospital Days All multisystem inflammatory syndrome in children (MIS-C) and COVID-19 hospitalizations (patients aged <21 years) across 31 states in 2021 by low vulnerability, representing the bottom 25% of the Centers for Disease Control and Prevention Social Vulnerability Index (SVI) data (A), and high vulnerability, representing the top 25% of the SVI (B). Days are risk adjusted for age, sex, and chronic conditions. Source: Agency for Healthcare Research and Quality Healthcare Cost and Utilization Project quarterly inpatient data and the SVI data. *P* values are for high and low comparisons within race and ethnicity and disease.

Since both Black and Hispanic children had disparities in long stays in Figure 1, in Figure 2 we show the examined findings on whether this disparity in length of stay may be exacerbated by socioeconomic factors, using the CDC SVI. Moving from the lowest to highest quartile of the SVI, Black children with MIS-C had their disparity worsen, with a 1-day (*P* = .01) increase in their stay, while White and Hispanic children saw no significant change. In contrast, with COVID-19, there was no increase.

## Discussion

Most MIS-C studies to date have been serial case studies. To our knowledge, this cross-sectional study is the first large-scale population-level study of all MIS-C and COVID-19 pediatric cases billed by the 4057 hospitals in 31 states in 2021. With this large data set, we were able to examine outcomes and use at their various stages of organ system failure. While the CDC reports an MIS-C death rate of 0.8% and 0.7% for pediatric COVID-19, we found that, when 6 or more organ systems were affected, the death rate increased to 5.8%% for MIS-C and to 17.2% for COVID-19.^23,24^ We also report what is, to our knowledge, the first analysis of AMEs in the treatment of MIS-C and COVID-19. These AMEs increased significantly over the number of organ systems involved for both MIS-C and COVID-19. Similarly, hospital length of stay doubled and costs tripled for MIS-C, while hospital length of stay tripled and costs increased to $100 000 for COVID-19 as the number of organ system complications increased.

With such similar outcome patterns between MIS-C and COVID-19, one may wonder whether severe MIS-C and COVID-19 cases are actually one and the same. However, we found starkly different patterns between the 2 diseases with respect to race. In particular, the percentage of patients with MIS-C who were Black doubled from 16.2% to 31.7% as the number of organ systems increased, while with COVID-19 there was no such change. Moreover, while we found disparities in 3 of 9 outcomes between Black and White children with COVID-19, this increased to 7 of 9 outcomes for MIS-C. Finally, while Javalkar et al^25^ found no association with the SVI on MIS-C outcomes in a case study of 43 patients with MIS-C, we found that Black patients with MIS-C had their length of stay increased by 1 day simply by living in the top 25% of socially vulnerable counties. Thus, severity of MIS-C for Black children was likely exacerbated by socioeconomic factors, even though this was not the case with COVID-19. Therefore, overall, MIS-C cases were fundamentally different from COVID-19 cases. Moreover, our results corroborate previous studies (Stierman et al^17^ and Payne et al^14^) that show MIS-C disparities in hospitalization rates go beyond SARS-2-CoV infection rate disparities. We extend this by showing in this study that MIS-C disparities extend even further to outcomes, unlike COVID-19.

Another advantage of our large data set is that it captures most community hospitals in the 31 states examined and all their billed MIS-C cases. In contrast, most MIS-C studies to date have used CDC surveillance data, which are based on data voluntarily reported by hospitals to state health departments. It is possible that many hospitals did not report their MIS-C cases. In our review of what Florida hospitals reported to their department of health, many counties did not report any cases.^26^ In 2021 Q1, only 37 MIS-C cases were reported by hospitals to the department of health, compared with the 190 billed MIS-C cases in our HCUP data over all counties in Florida (see eTable 2 in Supplement 1). Overall, our AHRQ HCUP data capture about twice as many cases of MIS-C than are reported to the CDC (eTable 2 and eFigure 2 in Supplement 1).^21,23^ Underreporting has been seen before with other types of voluntary reporting. For example, the CDC surveillance of *Clostridioides difficile* infections estimated 223 900 hospitalizations with *C difficile* nationally in 2017.^27^ The HCUP data reported 336 600 hospitalizations with *C difficile*.^28^

### Limitations

This study has limitations. A limitation of large all-payer billing data is that cases have not been adjudicated by having a medical team review the medical records. Thus, there may be some cases billed as MIS-C that do not fit the CDC actual definition on closer inspection of the medical record. In Stierman et al,^17^ 13% of adjudicated MIS-C cases were found to not adhere to the CDC definition of MIS-C. It is possible that some of our billing data on MIS-C cases might similarly not fit the CDC definition. However, also by adjudication one might find many more MIS-C cases that were not diagnosed. To evaluate this potential, we applied the CDC definition of MIS-C to our data (eAppendix 2 in Supplement 1) (fever, ≥2 organs affected, laboratory test result indicating inflammation, COVID-19 associated). We estimated that there may be 2213 extra MIS-C cases in our data. Half of these cases were a result of complications due to chemotherapy treatment for cancer and not MIS-C. This leaves about 1039 inflammation cases that could potentially be MIS-C. Taking these false-negatives into account and since only 9% of our hospital sample received a diagnosis of MIS-C, compared with 47% for COVID-19, we suspect that there may be many more MIS-C cases that were undiagnosed in our data. Another limitation with billing data is that there may be variation in how hospitals document organ dysfunction and AMEs. However, given that most of the hospitals reporting MIS-C and COVID-19 were teaching hospitals, this variation may be less of a concern in our sample.

Future efforts should focus on how to prevent both MIS-C and COVID-19 from progressing to multiple organ system dysfunction. This may entail studying the socioeconomic factors of these cases: lack of quick access to medical care; predisposing chronic conditions, such as asthma and obesity; exposure to environmental pollutants; and structural racism. There is also a need for more basic science research including genome-wide association studies and multiomics-based approaches linking between the genotype and phenotype of the disease among patients with MIS-C aimed at understanding the pathophysiologic characteristics of the disease and identifying groups at increased risk of severity progression.^29^

## Conclusions

In 2021, several patterns emerged for MIS-C. First, MIS-C hospitalizations were more common than previously reported, with about 17 MIS-C cases for every 100 COVID-19 cases. Second, MIS-C was more severe than commonly thought. This study found that the average outcome rates reported in past MIS-C studies can be misleading. The number of organ system dysfunctions matters, with outcome rates diverging significantly from the averages as multiple organ systems fail. Third, racial and ethnic disparities in outcomes emerged with MIS-C, but not so with COVID-19. Fourth, Black children in more vulnerable socioeconomic areas experienced greater severity in MIS-C outcomes but not for COVID-19.

## Notes

Supplement 1. **eAppendix 1.** Data Files **eAppendix 2.** Case Definition of MIS-C **eTable 1.** *ICD-10-CM* Codes for the Complications **eTable 2.** 2021 Q1 Reporting of MIS-C Cases: Surveillance Data vs Hospital Billing Data **eFigure 1.** Socioeconomic and Racial and Ethnic Disparities in Incidence of MIS-C and COVID-19 **eFigure 2.** 2021 CDC MIS-C Surveillance Data

Supplement 2.
Data Sharing Statement
