# Supplementary material for: Complications, Adverse Drug Events, High Costs, and Disparities in Multisystem Inflammatory Syndrome in Children vs COVID-19
Source: JAMA Netw Open. 2023 Jan 5;6(1):e2244975. doi: 10.1001/jamanetworkopen.2022.44975 (PMC9857408; doi:10.1001/jamanetworkopen.2022.44975)
Supplement: Supplement 1. — eAppendix 1. Data Files eAppendix 2. Case Definition of MIS-C eTable 1. ICD-10-CM Codes for the Complications eTable 2. 2021 Q1 Reporting of MIS-C Cases: Surveillance Data vs Hospital Billing Data eFigure 1. Socioeconomic and Racial and Ethnic Disparities in Incidence of MIS-C and COVID-19 eFigure 2. 2021 CDC MIS-C Surveillance Data [file jamanetwopen-e2244975-s001.pdf]

Supplementary Online Content

Encinosa W, Moon K, Figueroa J, Elias Y. Complications, adverse drug events, high costs, and disparities in multisystem inflammatory syndrome in children vs COVID-19. *JAMA Netw Open*. 2023;6(1):e2244975. doi:10.1001/jamanetworkopen.2022.44975

eAppendix 1. Data Files

eAppendix 2. Case Definition of MIS-C

eTable 1. *ICD-10-CM* Codes for the Complications

eTable 2. 2021 Q1 Reporting of MIS-C Cases: Surveillance Data vs Hospital Billing Data

eFigure 1. Socioeconomic and Racial and Ethnic Disparities in Incidence of MIS-C and COVID-19

eFigure 2. 2021 CDC MIS-C Surveillance Data

This supplementary material has been provided by the authors to give readers additional information about their work.

© 2023 Encinosa W et al. *JAMA Network Open.*

eAppendix 1. Data Files.

We use the following AHRQ Healthcare Cost and Utilization Project's (HCUP) 2021 quarterly inpatient data files:

Q1: TX, VA, VT;

Q1-Q2: AZ, CA, FL, MD, ME, MI, MS, OH, SC, WI, WA;

Q1-Q3: CO, GA, IA, KY, NJ;

Q1-Q4: CT, HI, KS, MN, MO, MT, ND, OR, PA, RI, SD, WV.

Hospital characteristics are from the AHA annual survey and population rates are from 2021 US Census estimates.

COVID-19-related hospitalizations and in-hospital deaths, defined by the discharge disposition, are identified by any-listed ICD-10-CM code of U07.1 (2019 novel coronavirus disease) on the discharge record. Per coding guidelines, the use of U07.1 is based on documentation by the provider or documentation of a positive COVID-19 test result.

Source: HCUP State Inpatient Databases (SID). Healthcare Cost and Utilization Project (HCUP). 2005-2009. Agency for Healthcare Research and Quality, Rockville, MD. https://www.hcup-us.ahrq.gov/sidoverview.jsp (accessed 1 Nov 2021).

© 2023 Encinosa W et al. *JAMA Network Open.*

eAppendix 2. Case Definition of MIS-C:

- An individual aged <21 years presenting with fever*, laboratory evidence of inflammation**, and evidence of clinically severe illness requiring hospitalization, with multisystem (≥2) organ involvement (cardiac, renal, respiratory, hematologic, gastrointestinal, dermatologic, or neurological); AND
- No alternative plausible diagnoses; AND
- Positive for current or recent SARS-CoV-2 infection by RT-PCR, serology, or antigen test; or exposure to a suspected or confirmed COVID-19 case within the 4 weeks prior to the onset of symptoms.

*Fever ≥38.0°C for ≥24 hours, or report of subjective fever lasting ≥24 hours
**Including, but not limited to, one or more of the following: an elevated C-reactive protein (CRP), erythrocyte sedimentation rate (ESR), fibrinogen, procalcitonin, d-dimer, ferritin, lactic acid dehydrogenase (LDH), or interleukin 6 (IL-6), elevated neutrophils, reduced lymphocytes, and low albumin.

Additional comments:
Some individuals may fulfill full or partial criteria for Kawasaki disease but should be reported if they meet the case definition for MIS-C.
Consider MIS-C in any pediatric death with evidence of SARS-CoV-2 infection.

Sources: Centers for Disease Control and Prevention. 2022. Information for Healthcare Providers about Multisystem Inflammatory Syndrome in Children (MIS-C). https://www.cdc.gov/mis/mis-c/hcp/index.html (accessed October 14, 2022). The May 2020 CDC Health Advisory on MIS-C: https://emergency.cdc.gov/han/2020/han00432.asp (accessed October 14, 2022).

© 2023 Encinosa W et al. *JAMA Network Open.*

eTable 1: *ICD-10-CM* Codes for the Complications

Complications	*ICD-10-CM dx and ICD-10-PCS procedure codes (all-listed diagnoses and procedures)*
Cardiovascular Complication	AMI I21, I22, I23<br>Heart failure I50, I73.9<br>Heart valve I34, I35, I36, I08<br>Arrest I46.9<br>Myocarditis I40, I41, I63,I51.4, I09.0, I01.2, A38.1, J11.82, J10.82<br>Arrhythmia I49, I47, I45.9, P29.1, P03.81<br>Cardiomyopathy I42, I25.5, I43, B33.24<br>Pericarditis/peri effusion I32, I30, M32.12, I313
	Coronary-artery aneurysm I25.41, I25.42, I72, I25.3, I28.1<br>Cardio Shock/SIRS R57.0, R652.1, R65.1
Respiratory Complication	Respiratory failure J80, J96, R09.2<br>Hemorrhage R04, P26<br>Bronchospasm J95.851<br>Lower respiratory infection, J98.01, J20, J21, J22, J93, J40 A01.03, A02.22, A20.2, A21.2, A22.1, A31.0, A37.01, A37.11, A37.81, A37.91,<br>A43.0, A48.1, A50.04, A52.72, A54.84, B01.2, B05.2, B06.81, B25.0, B37.1, B38.0, B38.1, B39.0, B39.1, B39.2, B58.3, B59, B77.81, J09.X1, J10.00, J10.01, J10.08, J11.00, J11.08,<br>J12.0, J12.1, J12.2, J12.3, J12.81, J12.89, J12.9, J13, J14, J15.0, J15.1, J15.20, J15.211, J15.212, J15.3, J15.4, J15.5, J15.6, J15.7, J15.8, J15.9, J16.0, J16.8, J17, J18.0, J18.1, J18.8, J18.9, J85.1
	ARDS J80
	Mechanical ventilation 5A1935, 5A1945, 5A1955, 5A0935, 5A0945, 5A0955
	Pleural effusion J90, J91
Neurological Complication	Hemiplegia G81,<br>Encephalitis G04, G05<br>Cerebral infarction I63, I67<br>Convulsion R56<br>Critical illness polyneuropathy G62.81, G72.81<br>Encephalopathy G93.40, G93.49, G93.41<br>Iritis/ uveitis H20.0, H44.13<br>Coma P91.5, R40.2,<br>Intracranial hemorrhage I61,<br>Demyelinating disorder G36, G37,<br>Acute disseminated encephalomyelitis G04.0
Hematological Complication	Embolism/thrombosis I82, I76, I26, I74<br>Defribination syndrome D65, P60<br>Anemia D64<br>Lymphopenia D72.81<br>Thrombocytopenia D69.3, D69.4, D69.5, D69.6
Renal Failure	N17, N99.0<br>Dialysis Z99.2, Z91.15, Z49.01, Z49.02, Z49.31, Z49.32 5A1D00Z, 5A1D60Z, 3E1M39Z
Gastrointestinal Complications	Dehydration (E860), nausea, vomiting, and abdominal pain (R10, R11), diarrhea/gastroenteritis (R19.7, P78.3, K59.1, K58.0, K52.9),<br>Liver Failure K72<br>Pancreatitis K85<br>Hepatitis/Hepatomegaly B15, B16, B17, B19, R16.2<br>Gallbladder Hydrops K82.1<br>Ascites R18
Musculoskeletal Complication	Arthritis or arthralgia M08, M13, M25.5, M26.62, M08.2<br>Myositis or myalgia M60, G72.41, M79.1

© 2023 Encinosa W et al. *JAMA Network Open.*

eTable 1. Continued: ICD-10 codes for the complications

Complications	*ICD-10-CM dx and ICD-10-PCS procedure codes (all-listed diagnoses and procedures)*
Mucocutaneous Complication	Conjunctivitis H10<br>Erythema L51-L54<br>Local edema R60.0<br>Desquamation R23.4<br>Rash R21
Adverse Medication Event	Glucocorticoids T38.0X5<br>Immunoglobulin T50.Z15<br>Anti-infective T37.1X5-T37.9X5<br>Diuretics T50.1X5-T50.2X5<br>Antibiotics T36.0X5-T36.9X5- T37.0X5<br>Unspecified T50.9X5<br>Aspirin T39.015<br>Antihypertensive, anticoagulant, antithrombotic, fibrinolysis, thrombolytic T46.5X5, T45.515, T45.7X5, T45.525, T45.605, T45.695, T45.615

© 2023 Encinosa W et al. *JAMA Network Open.*

eTable 2. 2021 Q1 Reporting of MIS-C Cases: Surveillance Data vs Hospital Billing Data

States with public Q1 reports	State Department of Health MIS-C Cases Reported to CDC	All Hospital Billed MIS-C Cases in HCUP
Washington	20	28
Minnesota	26	37
Michigan	36	64
Florida	37	190
Wisconsin	40	48
Georgia	170	225
California	370	581
CDC, National (50 States/ DC)	1,924	
HCUP, 31 States (77% of US)		2,715

.

Our HCUP data's 2,715 MIS-C hospitalizations in Q1 is a rate of 1.48 per 100,000 children per month. In eFigure 2, the CDC data records 1,924 MIS-C cases for the country in Q1, a rate of .75 per 100,000 children per month. The two rates differ at significance level p=.001.

Sources:
HCUP (authors' calculations). Centers for Disease Control and Prevention. 2022. Health Department-Reported Cases of Multisystem Inflammatory Syndrome in Children (MIS-C) in the United States https://covid.cdc.gov/covid-data-tracker/#mis-national-surveillance (accessed 13 Oct 2022).
Florida Department of Health. COVID-19: characteristics of cases in pediatric Florida residents <18 years old. http://ww11.doh.state.fl.us/comm/_partners/covid19_report_archive/pediatric-reports/pediatric_report_latest.pdf (accessed 13 Oct 2022).
Multisystem Inflammatory Syndrome in Children Associated with COVID-19 in Washington. Oct 11, 2022. https://doh.wa.gov/sites/default/files/2022-02/MultisystemInflammatorySyndromeChildrenCOVID19WA2020.pdf MIS-C Statistics. Sept, 2022 (accessed 15 Oct, 2022).
Minnesota Dept of Health. https://www.health.state.mn.us/index.html .
MIS-C Data and Reporting. Michigan DHHS. https://www.michigan.gov/coronavirus/stats/mis-c-reporting . MIS_C Data. Sept, 2022 (accessed 15 Oct, 2022)..
Wisconsin Department of Health Services. https://www.dhs.wisconsin.gov/disease/mis-c-data.htm . Multisystem Inflammatory Syndrome in Children (MIS-C) Data. Sept, 2022 (accessed 15 Oct, 2022)..
California Dept of Public Health. https://www.cdph.ca.gov/Programs/CID/DCDC/Pages/COVID-19/MIS-C-Data.aspx . Multi-system Inflammatory Syndrome in Children- Associated with COVID 19 (MIS-C). June 28, 2022 (accessed 15 Oct, 2022).
Georgia Dept of Health. https://dph.georgia.gov/document/document/mis-c-june-2022-data-report/download (accessed 15 Oct, 2022).

eFigure 1: Socioeconomic and Racial and Ethnic Disparities in Incidence of MIS-C and COVID-19

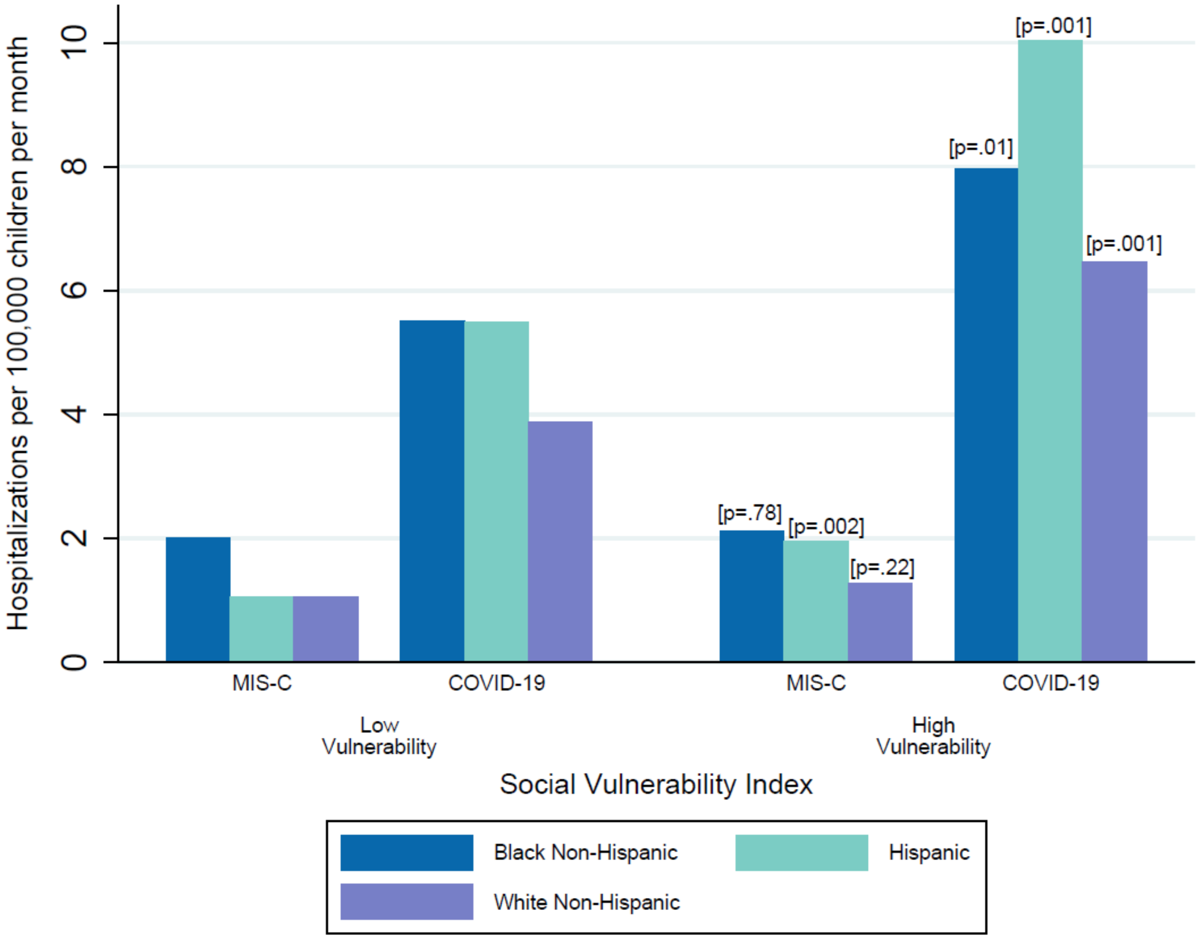

© 2023 Encinosa W et al. *JAMA Network Open.*

eFigure 2: 2021 CDC MIS-C Surveillance Data

Source: Centers for Disease Control and Prevention. 2022. Health Department-Reported Cases of Multisystem Inflammatory Syndrome in Children (MIS-C) in the United States https://covid.cdc.gov/covid-data-tracker/#mis-national-surveillance (accessed 13 Oct 2022).

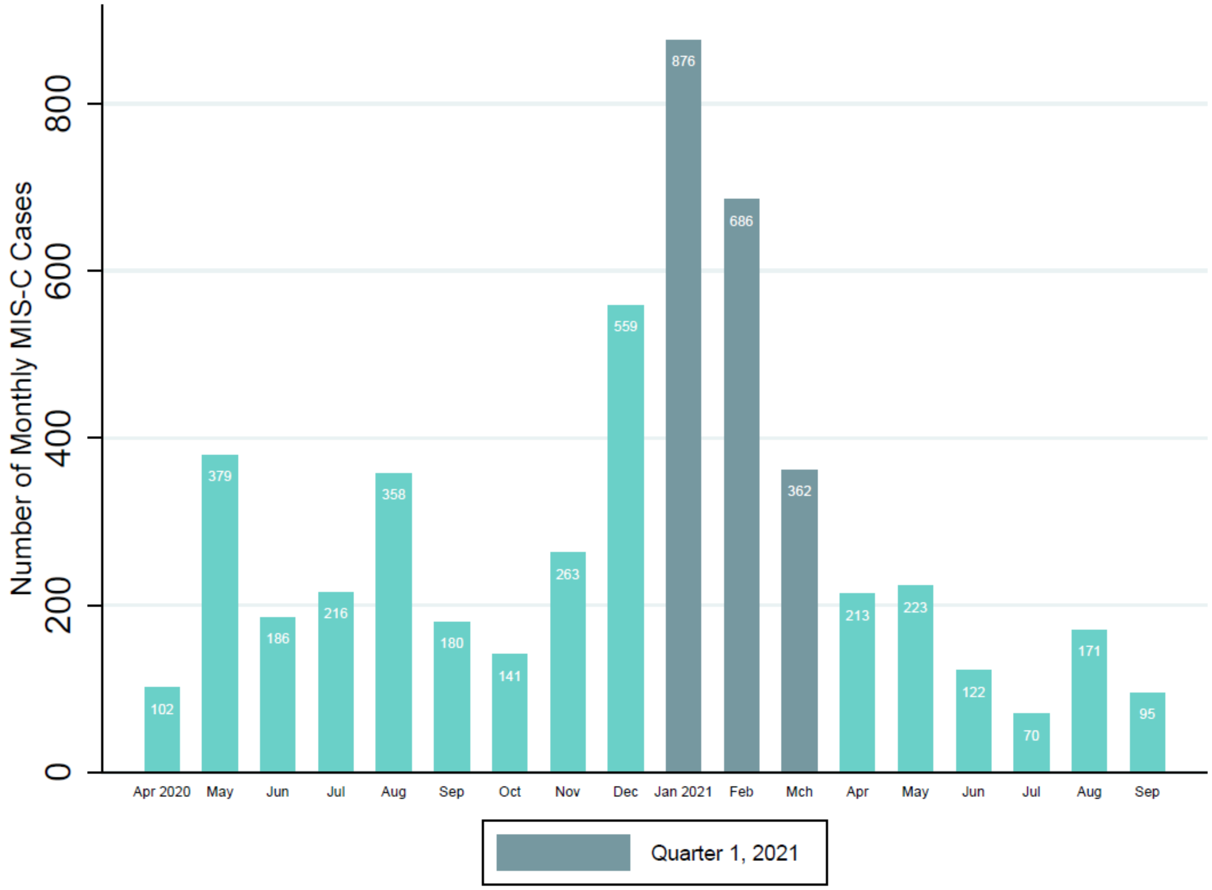

Notes: Monthly Multisystem Inflammatory Syndrome for Children (MIS-C) infection cases (age<21). Source: Authors' calculations using the CDC COVID Data Tracker, based on CDC surveillance data.

© 2023 Encinosa W et al. *JAMA Network Open.*
