# Supplementary material for: Complications, Adverse Drug Events, High Costs, and Disparities in Multisystem Inflammatory Syndrome in Children vs COVID-19
Source: JAMA Netw Open. 2023 Jan 5;6(1):e2244975. doi: 10.1001/jamanetworkopen.2022.44975 (PMC9857408; doi:10.1001/jamanetworkopen.2022.44975)
Supplement: Supplement 2. — Data Sharing Statement [file jamanetwopen-e2244975-s002.pdf]

Data Sharing Statement

Encinosa. Complications, Adverse Drug Events, High Costs, and Disparities in Multisystem Inflammatory Syndrome in Children vs COVID-19. *JAMA Netw Open*. Published January 05, 2023. doi:10.1001/jamanetworkopen.2022.44975

Data
Data available: No

Additional Information
Explanation for why data not available: The data used for this study were internal federal data from the AHRQ's Healthcare Cost and Utilization Project (HCUP). Publicly available data also are available through the HCUP Central Distributor: www.hcup-us.ahrq.gov/tech_assist/centdist.jsp.
